# A sialic acid-binding protein in *Toxoplasma gondii* contains a conserved globular domain in apicomplexan parasites

**DOI:** 10.1186/s13071-025-06845-5

**Published:** 2025-07-01

**Authors:** Xiaoyu Sang, Yize Liu, Yiwei Zhang, Ran Chen, Ying Feng, Ning Jiang, Qijun Chen

**Affiliations:** 1 Key Laboratory of Livestock Infectious Diseases, Ministry of Education, and Key Laboratory of Ruminant Infectious Disease Prevention and Control (East), Ministry of Agriculture and Rural Affairs, College of Animal Science and Veterinary Medicine, Shenyang Agricultural University, 120 Dongling Road, Shenyang, 110866 China; 2 Research Unit for Pathogenic Mechanisms of Zoonotic Parasites, Chinese Academy of Medical Sciences, 120 Dongling Road, Shenyang, 110866 China

**Keywords:** *Toxoplasma gondii*, Globular domain, Sialic acid

## Abstract

**Background:**

Apicomplexan protozoans employ an intricate invasion mechanism involving dynamic interactions with host cells, characterized by sequential secretion of adhesins and lectins. Our laboratory previously identified TgSABP1, a novel *Toxoplasma gondii* adhesin, demonstrating specific binding affinity for sialic acid (SA) receptors on host cell surfaces. However, the structural determinants governing SA recognition by this adhesin remain undefined.

**Methods:**

Three-dimensional structural predictions of TgSABP1 and homologous proteins were generated using AlphaFold2. Bio-layer interferometry (BLI) quantified the binding affinities between the recombinant proteins and ligands. Competitive BLI assays evaluated small molecules that potentially inhibit the TgSABP1–sialyllactose interactions. Molecular docking simulations employing AutoDock Vina software elucidated ligand-binding site interactions. In vitro invasion inhibition assays were performed to assess the therapeutic potential of lead compounds targeting TgSABP1 against *T. gondii* tachyzoites.

**Results:**

AlphaFold2 structural predictions revealed that TgSABP1 and its homologues contain a conserved globular domain (pLDDT > 90) with significant structural homology (with root-mean-square deviation [RMSD] < 4 Å) to a *Plasmodium falciparum* invasion-related protein PfIMP2 (PDB: 5LG9). BLI quantification demonstrated the micromolar binding affinities of the recombinant proteins for 3′-sialyllactose-polyacrylamide (PAA) and 6′-sialyllactose (6′SL)-PAA. Intriguingly, although recombinant TgSABP1 showed stronger lactose binding (*K*_D_ = 0.02 ± 0.01 M) compared to SA (*K*_D_ = 2.07 ± 0.45 M), only the latter exhibited an inhibition on the TgSABP1-6′SL-PAA interaction. Virtual screening of Food and Drug Administration (FDA)-approved compounds identified eltrombopag as a high-affinity molecule (ΔG_bind_ = −8.3 kcal/mol) targeting the SA-binding pocket in TgSABP1. Functional validation demonstrated that eltrombopag effectively blocked the TgSABP1/6′SL-PAA interaction and significantly decreased host cell invasion of *T. gondii* tachyzoites.

**Conclusions:**

Our study reveals a conserved globular domain of apicomplexan parasites as a novel SA-binding domain. Structural and functional characterization demonstrates its critical role in mediating TgSABP1-host cell interactions. Targeting this SA-binding pocket with eltrombopag effectively decreased *T. gondii* tachyzoite invasion, suggesting its therapeutic potential as an anti-invasion target. These findings not only elucidate a conserved mechanism underlying host receptor recognition in apicomplexans, but also establish a structural framework for the rational design of broad-spectrum inhibitors targeting invasion-related lectin domains.

**Graphical abstract:**

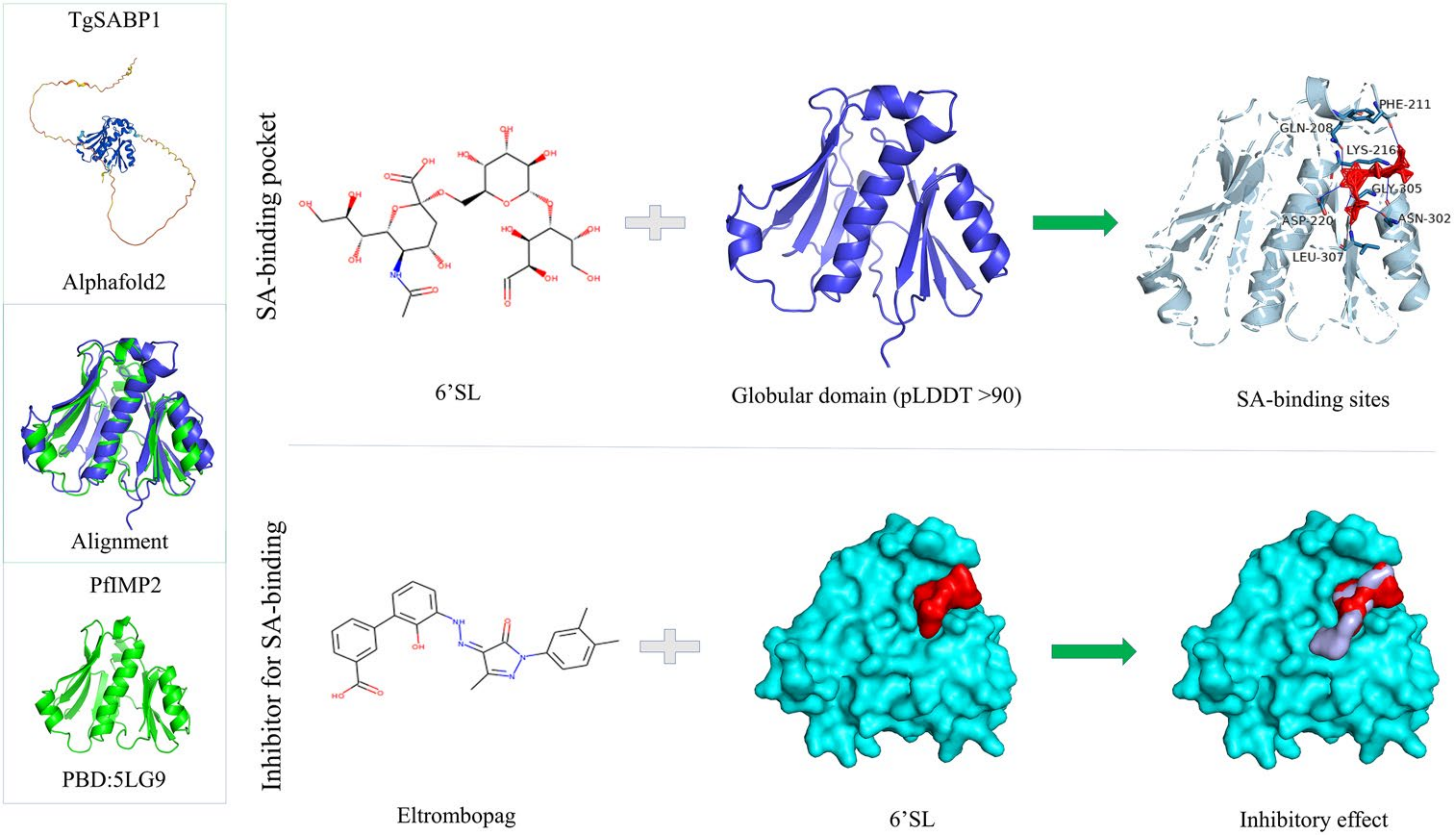

**Supplementary Information:**

The online version contains supplementary material available at 10.1186/s13071-025-06845-5.

## Background

The cellular surfaces of vertebrate species, including humans, are enveloped by a dense glycoconjugate matrix termed the glycocalyx. This evolutionarily conserved structure predominantly consists of sialylated glycoproteins and glycolipids, with terminal sialic acid (SA) residues serving as critical mediators of cellular recognition [1, 2]. SA predominantly occupies terminal positions on glycan chains through α-2,3 or α-2,6 glycosidic linkages to galactose (Gal) or *N*-acetylgalactosamine (GalNAc), forming stereochemical features that govern pathogen–receptor recognition. The specific linkage configuration of SA residues fundamentally influences viral tropism and pathogenesis. Comparative studies of influenza A viruses (IAVs) reveal a species-specific binding pattern: avian-origin hemagglutinin (HA) exhibits preferential affinity for α-2,3-linked SA receptors abundant on avian intestinal epithelia, while human-adapted HA variants predominantly recognize α-2,6-linked SA moieties, which are characteristic of human upper respiratory tract mucins [3]. This receptor dichotomy constitutes a major species barrier in interspecies transmission. Recent structural investigations have identified analogous mechanisms in coronaviruses. The Middle East respiratory syndrome coronavirus (MERS-CoV) spike protein demonstrates selective binding to α-2,3-linked SA derivatives, with markedly reduced affinity for α-2,6-linked counterparts [4]. Notably, SARS-CoV-2 employs a distinct recognition strategy through its receptor-binding domain (RBD), exhibiting preferential interaction with monosialylated gangliosides rather than simple SA-Gal/GalNAc conjugates. This molecular adaptation facilitates viral attachment to lipid raft microdomains, potentially enhancing membrane fusion efficiency during host cell entry [5]. 

Apicomplexan parasites employ sophisticated invasion mechanisms distinct from endocytosis utilized by viral or bacterial pathogens. This invasion paradigm requires orchestrated interactions between parasite-derived adhesins/lectins and host surface glycoconjugates. A hallmark of *Plasmodium* species lies in their erythrocyte binding-like (EBL) superfamily proteins, characterized by conserved extracellular cysteine-rich Duffy binding-like (DBL) domains not yet reported in other apicomplexan genera [6, 7]. Structural studies reveal these DBL domains mediate species-specific glycan recognition: *Plasmodium falciparum* erythrocyte-binding antigen 175 (EBA-175) selectively engages the α-2,3-sialylated *O*-glycan on glycophorin A (GPA), a critical determinant for erythrocyte tropism [8]. Parallel investigations demonstrate that PfEBA-140 binds to combinatorial N/O-linked sialylated glycan motifs on glycophorin C, suggesting functional diversification within the EBL family to exploit distinct erythrocyte surface architectures [9]. This ligand-receptor specificity not only governs erythrocyte invasion efficiency but may also influence host cell tropism. Comparative genomic analyses indicate that EBL protein expansion in *Plasmodium* reflects evolutionary adaptation to vertebrate erythrocyte surface diversity, providing a molecular framework for understanding host-parasite coevolution. 

Emerging evidence indicates that *Toxoplasma gondii* tachyzoites employ SA-dependent invasion pathways mediated by parasite-derived lectins dynamically secreted onto the apical membrane [10–12]. Biochemical characterization reveals microneme protein 1 (MIC1) as a pivotal SA-binding adhesin, marking the first identified member of the invasion-critical micronemal protein family [13]. Structural determination through X-ray crystallography demonstrates that TgMIC1 harbors a unique micronemal adhesive repeat (MAR) domain, exhibiting a preferred affinity for α-2,3-linked SA while showing limited binding capacity for α-2,6-linked isoforms [14, 15]. Phylogenomic analyses reveal that MAR domain-containing proteins are evolutionary innovations restricted to coccidian parasites (*Toxoplasma*, *Neospora*, and *Eimeria*), suggesting lineage-specific adaptation to SA-rich host niches [2]. Our recent work identified a novel SA-binding effector in *T. gondii*, named sialic acid-binding protein 1 (TgSABP1), localized on the tachyzoite surface and functionally implicated in host cell entry [16]. Intriguingly, TgSABP1 exhibits a divergent architecture containing a single IMP2_N domain (PF18590)—a structural motif first characterized in *Eimeria maxima* immune mapped protein 1 (IMP1) and conserved across apicomplexans [17, 18]. This finding raises critical questions about functional convergence: while IMP2_N domains are apicomplexan features, their co-option for SA recognition in *Toxoplasma* may represent a distinct characteristic compared to the coccidian-specific MAR domains. However, the defined interaction between the TgSABP1 protein and the SA receptor is still unclear and warrants further investigation.

In this study, AlphaFold2 structural prediction revealed a high-confidence globular domain in TgSABP1 (pLDDT > 90) with striking topological similarity to PfIMP2. This conserved globule domain across apicomplexan parasites conferred the SA-binding abilities of TgSABP1 and its homologues. The SA binding pocket of TgSABP1 was determined by molecular docking, and the compounds targeting this pocket effectively blocked the binding of recombinant TgSABP1 to sialyllactose polymer, significantly reducing the parasite invasion in vitro. Our findings deepen the understanding of the interaction between *T. gondii* lectin and host cell receptor, providing a molecular basis for screening anti-*T. gondii* drugs based on invasion-associated proteins.

## Methods

### Cell culture and parasite maintenance

Vero cells (ATCC CCL-81) were cultured in Dulbecco’s modified Eagle medium (DMEM; Servicebio, Wuhan, China) supplemented with 8% (v/v) heat-inactivated fetal bovine serum (FBS; Gibco) at 37 °C under a 5% CO₂ atmosphere. The *T. gondii* RH-RFP strain stably expressing red fluorescent protein was maintained by serial passages in confluent Vero cell monolayers under identical culture conditions.

### Molecular cloning and recombinant protein expression

Gene-specific primers (Additional file 1: Table S1) were designed to amplify coding sequences of *T. gondii* proteins (TgSABP1 [TGGT1_225940], TgIMP1 [TGGT1_293470], TgIMP2.1 [TGGT1_276930]) and *P. falciparum* proteins (PfIMP1 [PF3D7_1006000], PfIMP2 [PF3D7_0730400], PbIMP2 [PBANKA_0214500]) from complementary DNA (cDNA) using high-fidelity polymerase chain reaction (PCR). Amplified fragments were directionally cloned into pDEST-17 Gateway vector (Invitrogen, Carlsbad, CA, USA) for TgSABP1 and pET-28a ( +) vector (Novagen, Madison, WI, USA) for other targets. Recombinant plasmids were transformed into chemically competent *Escherichia coli* BL21(DE3) cells (TransGen Biotech, Beijing, China). His-tagged proteins were expressed by 0.5 mM isopropyl β-D-1-thiogalactopyranoside (IPTG) and induction at 16 °C for 18 h, and purified by nickle–nitrilotriacetic acid (Ni–NTA) affinity chromatography following established protocols [16].

### Structural modeling and molecular docking

Predicted tertiary structures of parasite proteins were generated using AlphaFold2 (v2.3.0) with default parameters, including TgSABP1, TgIMP2.1, TgIMP1, PbIMP2, and PfIMP1 (Additional file 1: Figure S1). Small molecule ligands (3′-sialyllactose, 6′-sialyllactose, SA, FDA-approved drugs) were retrieved from the ZINC20 database (v2022). Molecular docking simulations were performed in AutoDock Vina (v1.5.6) software with the following workflow:

1. Protein preparation: Remove water molecules > Add polar hydrogens > Assign Gasteiger charges
2. Ligand preparation: Detect rotatable bonds > Generate conformers
3. Docking: Flexible grid box (45.75 × 44.25 × 29.25 Å) > Exhaustiveness = 10

Binding affinities were ranked by calculated ΔG_bind_ values, with lower (more negative) values indicating stronger interactions [19]. Protein–ligand interactions were visualized using PyMOL (v2.5.4) software [20]. Oseltamivir and eltrombopag screened from the FDA databases were selected for further analysis based on preliminary docking results.

### Bio-layer interferometry (BLI) analysis on the affinity between recombinant proteins and sialylated oligosaccharides

Two sialylglycopolymers, Neu5Acα3′-Lac-Gly-PAA [polyacrylamide]-biotin (3′SL- PAA) and Neu5Acα6′-Lac-C2-PAA-biotin (6′SL-PAA), were purchased from GlycoNZ (Auckland, New Zealand) and are commonly used to identify the SA binding preference of pathogens [21, 22]. Binding kinetics between His-tagged recombinant proteins and immobilized sialylglycopolymers were quantified using an Octet K2 BLI system (Sartorius, Göttingen, Germany) following optimized protocols [23, 24]. The experimental workflow included ligand loading, protein dilution, and binding cycles. Briefly, streptavidin biosensors (Sartorius, Göttingen, Germany) were immersed in 2.4 μM 3′SL-PAA/6′SL-PAA solutions (phosphate-buffered saline [PBS], pH 7.2) until saturation (≥ 1.5 nm wavelength shift). Recombinant proteins were serially diluted in PBST-BSA buffer (PBS + 0.5% Tween 20 + 0.1% bovine serum albumin [BSA]) to different concentrations: TgSABP1 (1, 2, 4 μM for 3′SL-PAA and 1.25, 2.5, 5 μM for 6′SL-PAA), PfIMP2 (30, 50, 70 μM for 3′SL-PAA and 15, 30, 50 μM for 6′SL-PAA), PbIMP2 (5, 10, 20 μM for 3′SL-PAA and 6′SL-PAA), PfIMP1 (2.5, 5, 10 μM for 3′SL-PAA and 6′SL-PAA), TgIMP2.1 (0.625, 1.25, 2.5 μM for 3′SL-PAA and 6′SL-PAA) and TgIMP1 (0.75, 1.5, 3 μM for 3′SL-PAA and 6′SL-PAA). The binding tests included equilibration for 90 s in PBST-BSA, association for 90–120 s in protein solutions, dissociation for 60–90 s in PBST-BSA, and regeneration [3 × 20 s pulses in HCl (pH 0.5) followed by PBS neutralization]. Data parameters were analyzed using ForteBio Data Analysis software (v 9.0.0.14) in the average reference sensors’ subtraction method and 1:1 binding model for *K*_D_ calculations.

### Binding of recombinant PfIMP2 to human erythrocytes

The PfIMP2 coding sequence was cloned into pGEX-4 T-1 (Invitrogen, Carlsbad, CA, USA). Recombinant GST-PfIMP2 and GST control protein were expressed in *E. coli* BL21(DE3) (TransGen Biotech, Beijing, China) under 0.5 mM IPTG induction at 16 °C for 18 h, followed by purification via glutathione affinity chromatography (GE Healthcare, Chicago, IL, USA). The diluted GST-tagged PfIMP2 and GST protein (as negative control) were incubated with human red blood cells as previously described [16]. The cell pellets were washed three times with PBS and mixed with 5 × SDS loading buffer (Beyotime, Shanghai, China), and analyzed by western blot. For the immunofluorescence assay (IFA), human red blood cells on coverslips were incubated with 1 μM GST-tagged PfIMP2 and GST protein at room temperature. After three washes with PBS, each coverslip was fixed with 4% paraformaldehyde (PFA) and detected with anti-GST monoclonal antibody (Beyotime, Shanghai, China), followed by Alexa Fluor 488-Labeled Goat Anti-Mouse IgG (H+L) (Beyotime, Shanghai, China). The fluorescence signal of cells on the coverslips was observed using a fluorescence microscope (Leica DM4 B, Wetzlar, Germany).

### BLI-based affinity quantification of small molecules with TgSABP1

Binding kinetics between TgSABP1 and carbohydrates were analyzed using an Octet K2 BLI system. Briefly, NTA biosensors (Sartorius, Göttingen, Germany) were loaded with 1 μM His-tagged TgSABP1 (PBS, pH 7.2) until saturation (≥ 4 nm shift). Small molecules were diluted to different concentrations in PBST-BSA, including SA (0.5, 1, 2, and 4 mM), lactose (5, 10, 20, and 40 mM), oseltamivir phosphate (6.25, 12.5, 25, and 50 mM), and eltrombopag (10, 20, 30, 40, and 50 μM). The binding test included equilibration in PBST-BSA for 90 s, association in diluted carbohydrate solutions for 90 s, and dissociation in PBST-BSA for 60 and 120 s. The binding kinetic parameters were analyzed by ForteBio Data Analysis software (v9.0.0.14) in the double reference sensors subtraction method (buffer + unloaded sensor baselines) and steady-state affinity model for *K*_D_ determination. Results are expressed as the mean ± SD from three independent replicates.

### Competitive binding analysis of TgSABP1 inhibitors via BLI

To evaluate small molecule-mediated inhibition of TgSABP1-6′SL-PAA interaction, streptavidin biosensors pre-loaded with 2.4 μM 6′SL-PAA until saturation (threshold ≥ 1.5 nm shift) were subjected to competitive binding assays on an Octet K2 system. Four test compounds were diluted to different concentrations in PBST-BSA, including SA (5, 10, and 20 mM), lactose (25, 50, and 100 mM), oseltamivir phosphate (30 and 60 mM), and eltrombopag (5 and 50 μM). The His-tagged TgSABP1 protein was diluted to 5 μM with different concentrations of compounds and incubated for 15 min at room temperature. The competition binding test included equilibration for 90 s in the compound solution, association for 60–180 s in the pre-treated protein solution, dissociation for 60–90 s in the compound solution, and regeneration [3 × 20 s pulses in HCl (pH 1.0) followed by PBS neutralization]. Data parameters were analyzed by ForteBio Data Analysis software (v 9.0.0.14) using the average reference sensors’ subtraction method and 1:1 binding model. All experiments were repeated in triplicate.

### Cytotoxicity evaluation of eltrombopag to Vero cells

Vero cells were seeded in 96-well plates at 8000 cells/well and cultured to 90% confluence in DMEM with 8% FBS at 37 °C under 5% CO₂. The cells were treated with eltrombopag at concentrations of 5, 10, 20, or 40 μM in DMEM with 2% FBS. Cells incubated in DMEM containing the same amount of dimethyl sulfoxide (DMSO) used in the drug treatments served as a negative control. The blank control group consisted of cells with DMEM/2% FBS. After 2 h or 24 h of treatment with the compound, all cells were gently washed with PBS once and inoculated with DMEM containing 2% FBS for culturing 24 or 4 h, respectively. Then, the culture medium was removed, and all cells were incubated with DMEM containing 2% FBS and 10% CCK-8 reagent (Beyotime, Shanghai, China) for 1 h. The microplate reader (Hangzhou Allsheng Instruments, Hangzhou, China) was used to detect absorbance of cells at 450 nm. The cell survival rates were calculated with the following formula: cell survival rate (%) = (absorbance of drug treatment group–absorbance of free DMEM) / (absorbance of blank control group − absorbance of free DMEM) × 100%. Five independent experiments were performed as previously described [25].

### Evaluation of eltrombopag-mediated inhibition of *T. gondii* host cell invasion

The anti-invasive activity of eltrombopag against *T. gondii* was assessed using a fluorescence-based invasion assay with modifications to the established protocols [16, 25]. *Toxoplasma gondii* RH strains stably expressing red fluorescence protein were added to 2 × 10^5^ Vero cells (multiplicity of infection [MOI] of 1:1) pre-treated with the drug or the equivalent volume of DMSO for 1 h. One hour after the parasites were added, the parasites were removed and the cells were gently washed with PBS three times, then incubated with DMEM containing 2% FBS. After 16 h of cultivation, the cells were fixed with 4% PFA and stained with DAPI (Beyotime, Shanghai, China). The numbers of parasitophorous vacuoles (PVs) and host cells were counted in a total of 20 randomly selected fields under a fluorescence microscope (Leica DM4B, Wetzlar, Germany). Invasion efficiency = (number of PVs/number of DAPI^+^ cells) × 100%. Meanwhile, the number of parasites in each vacuole was determined. Seventy vacuoles from each coverslip were counted across at least three biological replicates. Intra-vacuolar proliferation was expressed as the mean number of parasites per PV.

### Statistical analysis

All data were analyzed using GraphPad Prism 5.0 (GraphPad Software, Inc., USA). The mean ± standard deviation (SD) was determined using at least three biological replicates. Statistical significance was defined as *P* < 0.05.

## Results

### A highly credible globular domain was identified in the SA-binding proteins of *T. gondii* and *Plasmodium* parasites

**Fig. 1:**
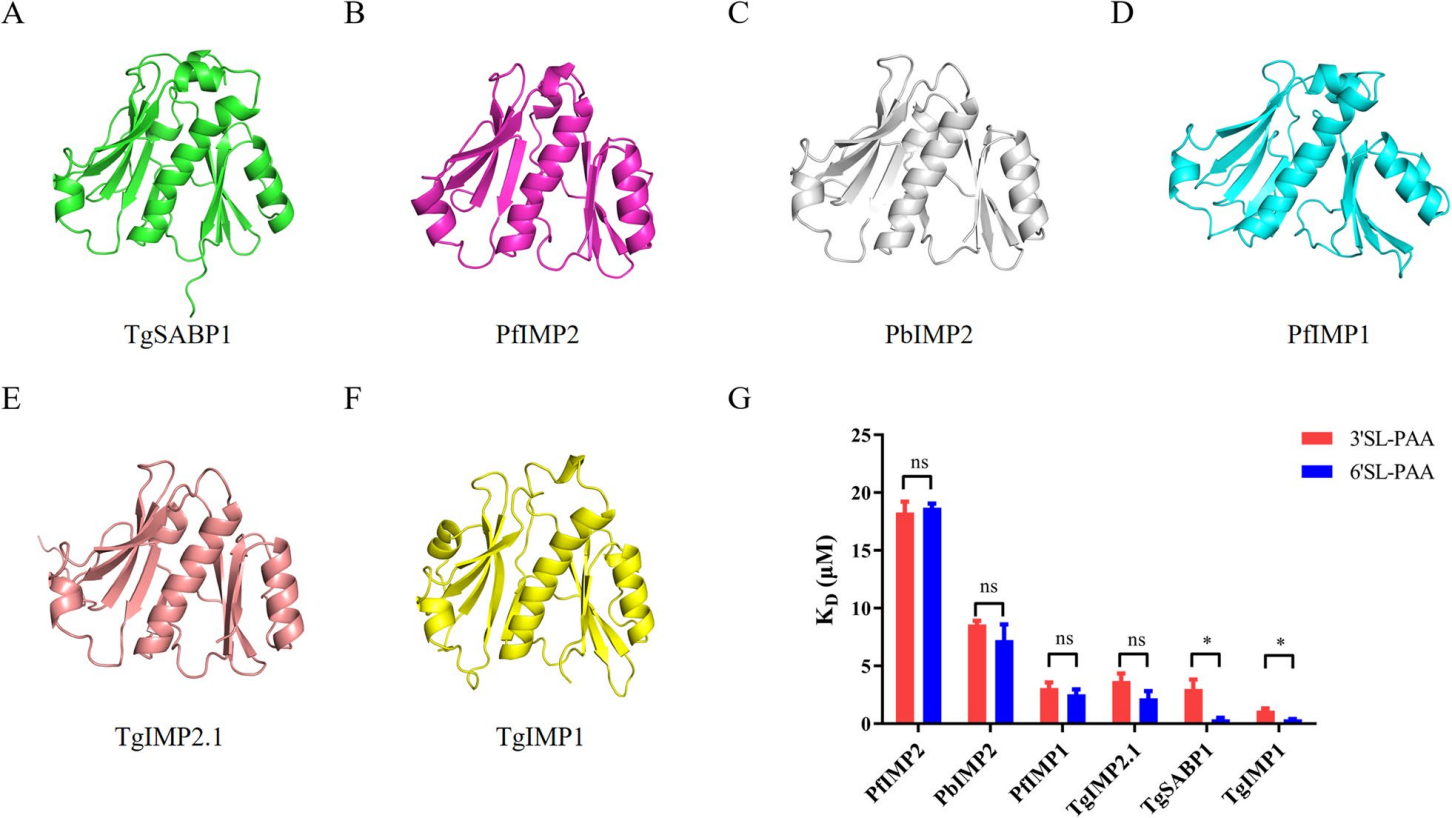
Analysis of three-dimensional (3D) structures of TgSABP1 and its homologues in *T. gondii* and *Plasmodium* parasites and their binding affinity to sialylglycopolymers. **A**–**F** A credible globular domain was present in the structures of TgSABP1, PfIMP2, PbIMP2, PfIMP1, TgIMP2.1, and TgIMP1. **G** The affinities of the recombinant proteins to 3′SL-PAA (red) and 6′SL-PAA (blue) were determined by BLI assay. *K*_D_ value represents the equilibrium dissociation constant. ns, no significant difference, *P < 0.05 (unpaired Student’s *t*-test). All data are presented as the means ± SD (*n* = 3)

AlphaFold2 predictions revealed a high-confidence globular domain (pLDDT > 90) in TgSABP1 (Fig. 1A), exhibiting significant structural homology to that of PfIMP2 (PDB: 5LG9; Fig. 1B and Additional file 1: Figure S1) with a root-mean-square deviation [RMSD] of 2.329 Å. Conserved topological features were observed in predicted models of *Plasmodium* homologues PbIMP2, PfIMP1, and *T. gondii* paralogues TgIMP2.1, TgIMP1 (Fig. 1C–F and Additional file 1: Figure S1), with RMSD values of 2.011 Å, 3.459 Å, 1.547 Å, and 2.823 Å, respectively. To evaluate their SA-binding abilities, recombinant His-tagged proteins were expressed in *E. coli* (Additional file 1: Figure S2) and subjected to BLI against sialylglycopolymers. All proteins bound to 3′SL-PAA and 6′SL-PAA with micromolar affinity (Fig. 1G and Additional file 1: Figure S3). Notably, recombinant TgSABP1 and TgIMP1 possessed significantly stronger binding ability to 6′SL-PAA than 3′SL-PAA (*P* < 0.05).

### The recombinant PfIMP2 binds to human erythrocytes

**Fig. 2:**
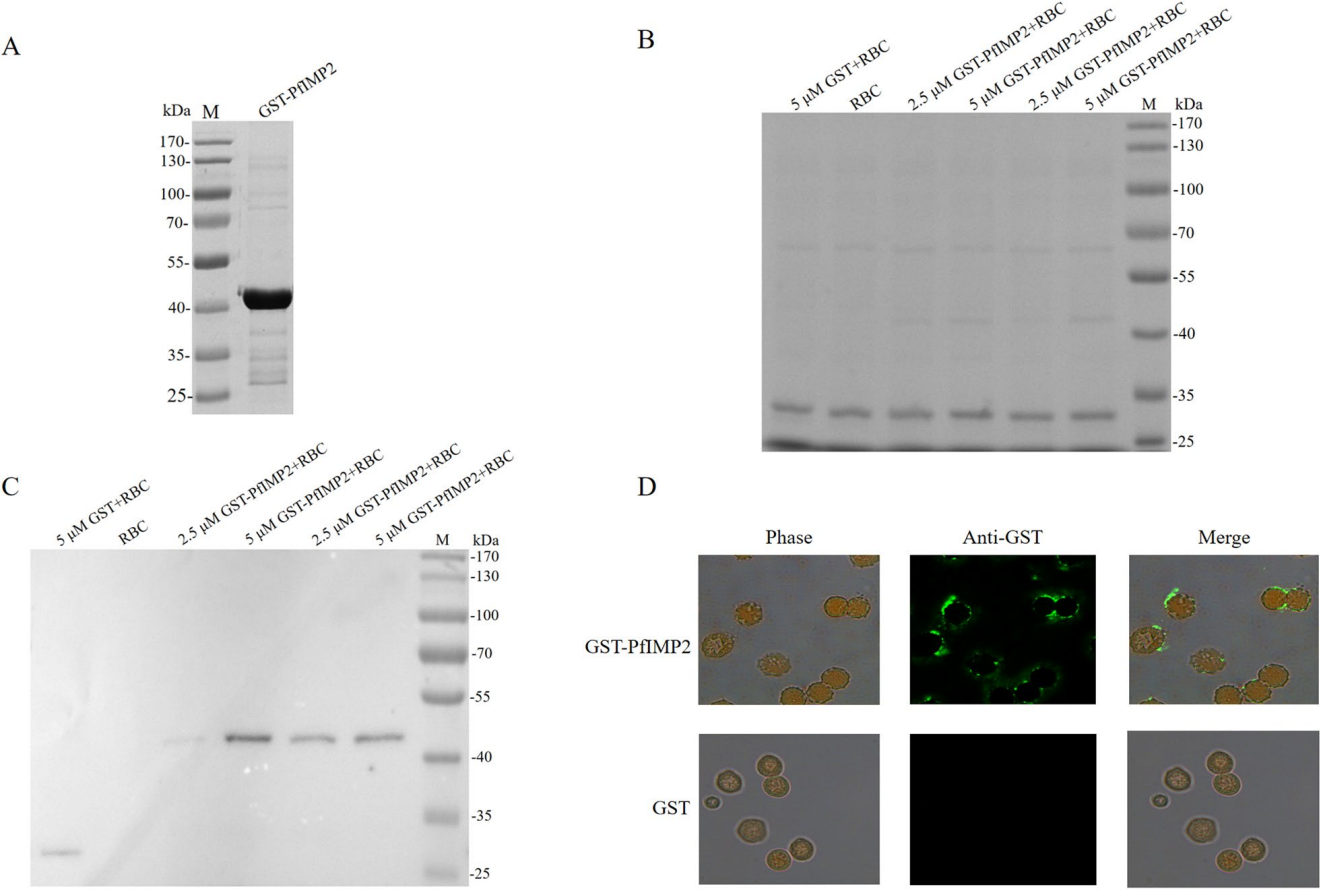
Specific binding of recombinant PfIMP2 to human erythrocytes. **A** Coomassie-stained SDS-PAGE of purified GST-tagged PfIMP2 (~ 44 kDa). **B** SDS-PAGE showing the same amounts of proteins were loaded in the gel after RBC-binding. **C** Western blot showing the binding of GST-PfIMP2 to RBC in 2.5 and 5 μM concentrations with two replicates. **D** Immunofluorescence micrographs demonstrating specific binding of GST-tagged PfIMP2 to human erythrocyte, scale bar = 20 μm. Images representative of three biological replicates

Given the high structural homology between TgSABP1 and PfIMP2**,** and the peak expression of PfIMP2 during the schizont stage of *P. falciparum* [26], we further evaluated the binding capacity of recombinant PfIMP2 to human red blood cells (RBCs). Recombinant GST-tagged PfIMP2 was expressed in *E. coli* and purified via glutathione affinity chromatography (Fig. 2A). Sodium dodecyl sulphate–polyacrylamide gel electrophoresis (SDS-PAGE), western blot, and erythrocyte adhesion assays revealed that the GST-tagged PfIMP2 bound to RBC in a concentration-dependent manner, whereas the GST protein alone did not (Fig. 2B, C, and D).

### Competitive inhibition of SA on TgSABP1-6′SL-PAA interactions

**Fig. 3:**
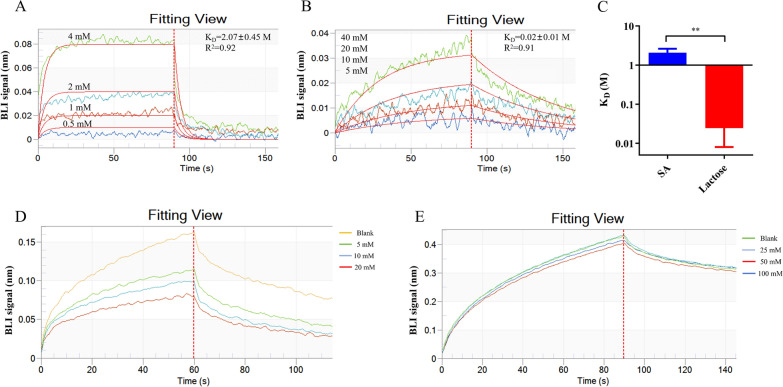
Inhibition analysis of SA on the binding of the recombinant TgSABP1 to 6′SL-PAA with lactose as a control. **A**, **B** Real-time binding sensograms for SA (**A**) vs. lactose (**B**). The *K*_D_ value represents the equilibrium dissociation constant. Data are presented as means ± SD (*n* = 3). The R^2^ value represents the fitting degree of the kinetic curve. **C** Comparative *K*_D_ values of SA and lactose with TgSABP1. **P < 0.01 (unpaired Student’s *t*-test). **D**, **E** Competitive binding profiles with SA (**D**) and lactose (**E**) in TgSABP1-6′SL-PAA interactions

As the ligand of 6′SL-PAA consists of SA and lactose by an α-2,6 glycosidic linkage, the binding abilities of TgSABP1 to both SA and lactose were further explored. BLI revealed distinct binding profiles and affinities of His-tagged TgSABP1 toward SA and lactose (Fig. 3A, B). The *K*_D_ values for the recombinant TgSABP1 to SA and lactose were determined to be 2.07 ± 0.45 M and 0.02 ± 0.01 M, respectively. These results indicated that the recombinant protein exhibited a greater affinity for lactose compared to SA, as evidenced by the lower *K*_D_ value for lactose (Fig. 3C). However, only SA exhibited the inhibition on the binding of recombinant TgSABP1 to 6′SL-PAA in a concentration-dependent manner, while lactose did not (Fig. 3D, E).

### Computational modeling of TgSABP1-carbohydrate interactions

**Fig. 4:**
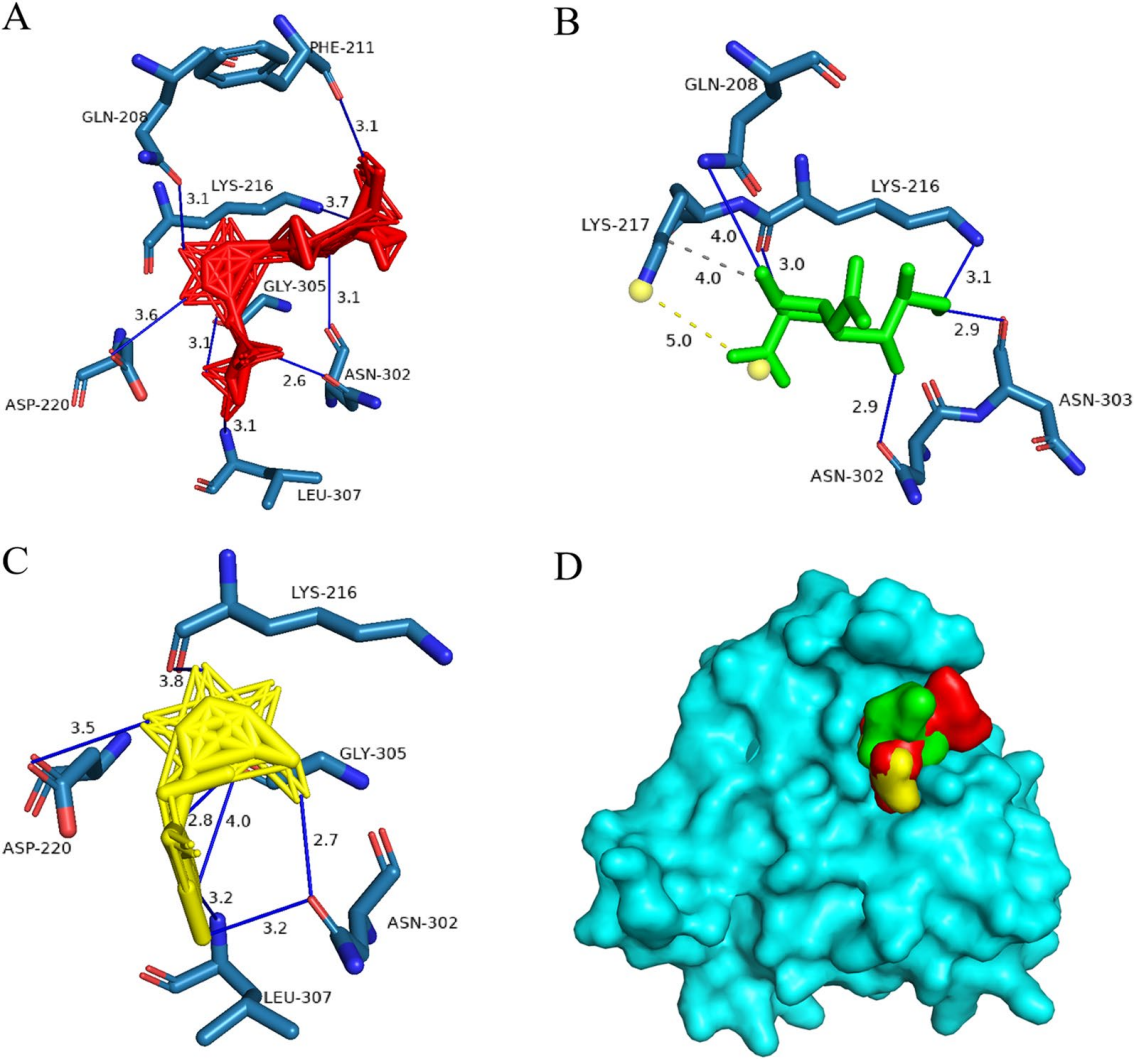
Molecular docking analysis of TgSABP1–SA interactions. **A** 6′SL (red sticks) forms eight hydrogen bonds (blue lines) with seven residues in TgSABP1. **B** SA (green sticks) binds to the TgSABP1 with one salt bridge (yellow dashes), one hydrophobic interaction (gray dashes) interaction, and five hydrogen bonds (blue lines). **C** Lactose (yellow sticks) forms seven hydrogen bonds (blue lines) with five residues of TgSABP1. **D** Structural overlap showing the relative positions of 6′SL (red), SA (green), and lactose (yellow) on the surface of TgSABP1 (cyan)

Protein–ligand interactions were simulated using AutoDock Vina software with the predicted globular domain (residues 122–313) of TgSABP1 as the receptor. The ΔG_bind_ between 6′SL and TgSABP1 was −6.7 kcal/mol and seven residues (Gln208, Phe211, Lys216, Asp220, Asn302, Gly305 and Leu307) were involved in eight hydrogen bonds with this molecule (Fig. 4A). SA formed five hydrogen bonds with four residues (Gln208, Lys216, Asn302 and Asn303) of TgSABP1, one salt bridge and one hydrophobic interaction with Lys217, yielding a ΔG_bind_ of −5.2 kcal/mol (Fig. 4B). At a ΔG_bind_ of −5.9 kcal/mol, lactose interacted with five residues (Lys216, Asp220, Asn302, Gly305, and Leu307) of TgSABP1 via seven hydrogen bonds (Fig. 4C). As the position of SA was spatially above the lactose, TgSABP1-SA interaction may create specific steric hindrance that prevents its binding to 6'SL (Fig. 4D). This spatial interference correlates with experimental inhibition data.

### High-throughput virtual screening identifies potent inhibitors of TgSABP1-6′SL interactions

**Fig. 5:**
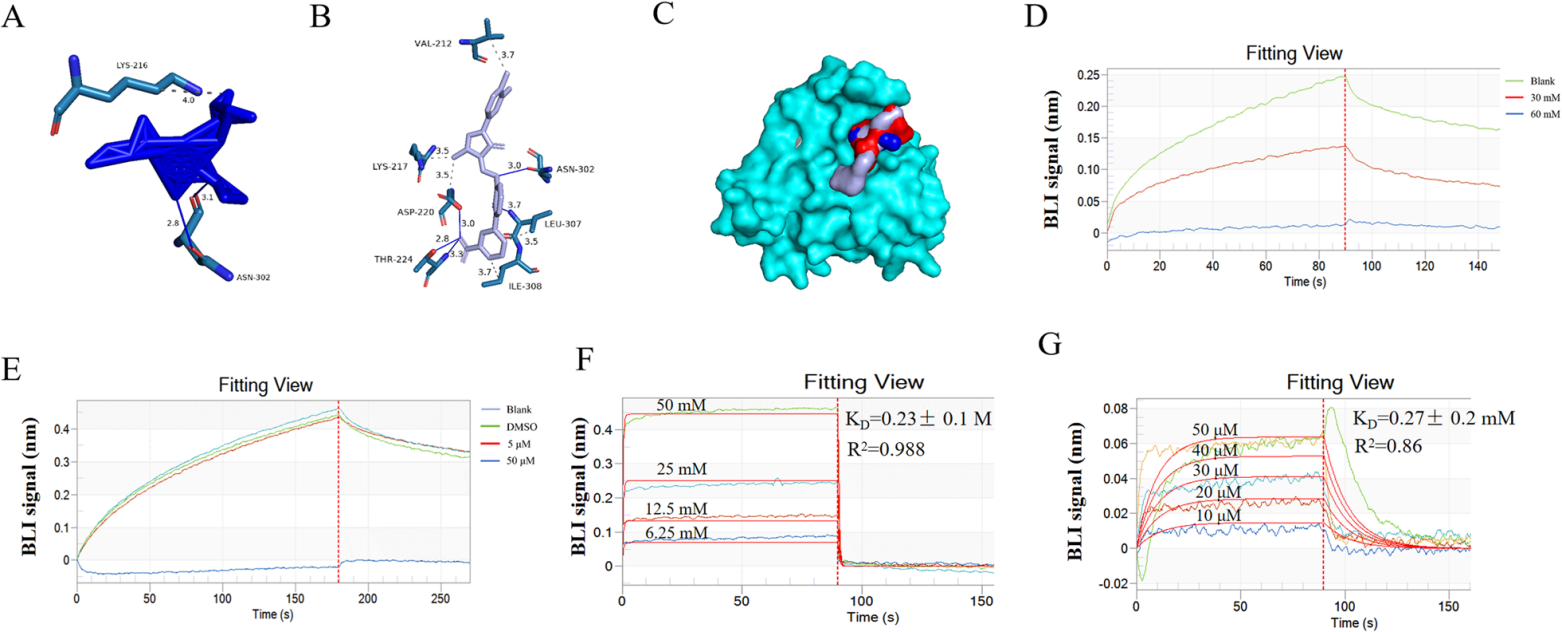
Docking and interaction validation between oseltamivir, eltrombopag, and TgSABP1. **A** Oseltamivir (blue) forms two hydrogen bonds (blue) and one hydrophobic interaction (gray dashes) with TgSABP1. **B** Eltrombopag (gray) formed five hydrogen bonds (blue lines) and five hydrophilic interactions (gray dashes) with TgSABP1. **C** Structural overlap showing the relative positions of 6′SL (red), oseltamivir (blue), and eltrombopag (light blue) on the surface of TgSABP1 (cyan). **D**, **E** Competitive binding profiles with oseltamivir phosphate (**D**) and eltrombopag (**E**) on TgSABP1-6′SL-PAA interactions. **F**, **G** Real-time binding sensograms for oseltamivir phosphate (**F**) and eltrombopag (**G**) with recombinant TgSABP1. The *K*_D_ value represents the equilibrium dissociation constant. The *R*^2^ value represents the fitting degree of the kinetic curve. Data are presented as means ± SD (*n* = 3)

The FDA-approved drug library containing 1606 compounds was virtually screened against the 6′SL-binding pocket of TgSABP1 using AutoDock Vina software, which identified 138 hits with predicted ΔG_bind_ <  − 6.7 kcal/mol. Among these, eltrombopag generated the lowest ΔG_bind_ of −8.3 kcal/mol. Notably, as an SA analogue, oseltamivir acquired the ΔG_bind_ of −4.8 kcal/mol, forming two hydrogen bonds with Asn302 and one hydrophobic interaction with Lys216 (Fig. 5A). Eltrombopag formed five hydrogen bonds with four residues (Asp220, Thr224, Asn302, and Leu307) and five hydrophilic interactions with other five residues (Val212, Lys217, Asp220, Leu307, and Ile308) on TgSABP1 (Fig. 5B). This indicated that the two compounds may exert similar steric hindrance effects on the TgSABP1/6′SL interaction as SA did (Fig. 5C). Further, 60 mM oseltamivir phosphate and 50 μM eltrombopag significantly blocked the binding of recombinant TgSABP1 to 6′SL-PAA (Fig. 5D, E). The *K*_D_ values of oseltamivir phosphate and eltrombopag with TgSABP1 were 0.23 ± 0.1 M and 0.27 ± 0.2 mM, respectively (Fig. 5F, G).

### Eltrombopag effectively inhibited *T. gondii* host cell invasion

**Fig. 6:**
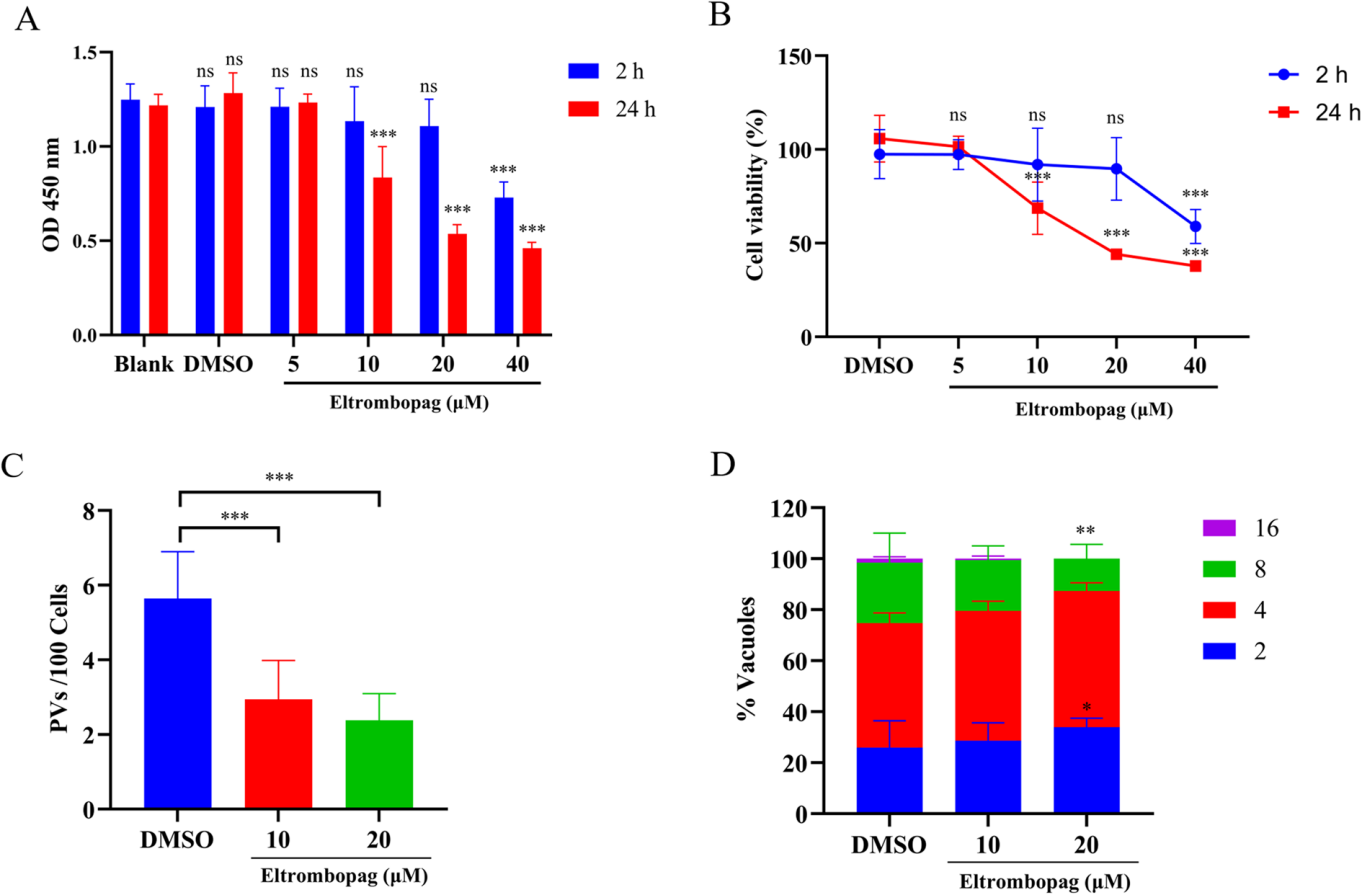
Cytotoxicity and anti-invasion activity of eltrombopag against *T. gondii*. **A** Cytotoxicity of eltrombopag on Vero cells after 2 h and 24 h exposure was detected by CCK-8 assay. **B** Cell viability curves of eltrombopag on Vero cells exposed to eltrombopag for 2 h and 24 h. **C** Invasion efficiency quantification of eltrombopag was calculated through PVs/DAPI^+^ cells. **D** The number of parasites per vacuole was determined in the eltrombopag-treated and DMSO groups. All data are expressed as the mean ± SD (*n* = 5–7). One-way analysis of variance (ANOVA) test for panels **A**–**C** and two-way ANOVA test with Dunnett’s multiple comparisons test for panel **D**. ns, not significant, *P < 0.05, **P < 0.01, ***P < 0.001

Eltrombopag, a thrombopoietin receptor agonist, has been indicated for the treatment of immune thrombocytopenia [27]. To assess its cytotoxicity, Vero cells were treated with eltrombopag (5–40 μM) for 2 h and 24 h, respectively. For 2-h treatment, compared to the negative control (only DMSO-treated), 40 μM eltrombopag showed significant cell growth inhibition, but not with lower concentrations. For the 24-h treatment, only the 5 μM eltrombopag did not show cytotoxicity to the cells (Fig. 6A, B). In the anti-invasion assay, compared with DMSO control, eltrombopag at both 10 μM and 20 μM significantly decreased host cell invasion by *T. gondii*, with the inhibition rates of 47.8% and 57.7%, respectively (Fig. 6C). The IC_50_ value of eltrombopag for anti-invasion of *T. gondii* was calculated as 11.62 μM. Compared with the DMSO control, the replication of invaded parasites was significantly impaired in the medium containing 20 μM eltrombopag (Fig. 6D).

## Discussion

Sialic acid, a terminal carbohydrate moiety ubiquitously expressed on the surface of vertebrate cells, serves as a critical recognition marker for host cell invasion by apicomplexan parasites, including *T. gondii* and *Plasmodium* spp. [10, 28]. While canonical SA-binding adhesins, such as TgMIC1 (via MAR domains) [14] and PfEBA-175 (via DBL domains) [29], exhibit relatively conserved structural architectures, our study identifies TgSABP1 as an SA-binding protein with a novel and distinct feature. Bioinformatic analysis reveals that TgSABP1 contains an IMP2_N domain, a structural motif first characterized in *E. maxima* IMP1 [17, 18, 30, 31]. As one of the IMP1 homologues, the three-dimensional (3D) structure of PfIMP2 has been resolved as a novel globular structure [32]. AlphaFold2 modeling demonstrates that the IMP2_ domain of TgSABP1 folds into a globular conformation (pLDDT > 90) with striking structural homology to the crystallized PfIMP2 (PDB: 5LG9). Carbohydrate microarrays showed that TgMIC1 preferred to α-2,3- over α-2,6-linked SA [15], while PfEBA-175 specifically recognizes α-2,3-linked SA presented on the glycophorin A of the erythrocyte [8]. In the present study, we demonstrated that TgSABP1 exhibits binding abilities for two kinds of sialyllactoses and has preferential binding to α-2,6-linked SA. The homologues of TgSABP1 in *T. gondii* and *Plasmodium* spp. all exhibited similar SA-binding abilities, which are likely mediated by the conserved globular domain across apicomplexan protozoa. Additionally, recombinant PfIMP2 exhibited a specific binding affinity for the surface of the human erythrocyte, which has abundant sialic acid receptors. This collective evidence establishes the IMP2_N globular domain as a novel SA-recognition module conserved across apicomplexans, distinct from MAR and DBL domains.

Our previous investigations revealed that SA effectively inhibited the interaction between recombinant TgSABP1 and SA-agarose matrix [16]. Building upon these findings, the current study demonstrates that SA specifically suppresses the binding of recombinant TgSABP1 to 6′SL-PAA through a competitive mechanism. Notably, despite exhibiting higher binding affinity for TgSABP1 compared to SA (Fig. 3C), lactose displayed little inhibitory efficacy on the protein’s interaction with 6′SL-PAA (Fig. 3D, E). Molecular docking analysis revealed that SA occupies a distinct spatial position above the lactose-binding site within the TgSABP1 structure. This SA-specific binding pocket, formed by eight residues including Gln208, Ala213, Lys216, Lys217, Asp220, Asn302, Asn303, and Gly305 (Additional file 1: Figure S4), appears critical for mediating SA-dependent receptor recognition.

To explore more effective inhibitors for the SA-binding of TgSABP1, we docked 1606 compounds in the FDA database with the target protein using Autodock Vina software and chose oseltamivir and eltrombopag for further analysis. Consistent with its established mechanism as an anti-influenza therapeutic agent targeting viral neuraminidase [33], oseltamivir phosphate demonstrated dose-dependent inhibition of TgSABP1-6′SL-PAA interaction in our system, mirroring that of SA’s inhibitory profile. Furthermore, we identified eltrombopag, a compound previously characterized as binding the DNA-interaction domain of *Salmonella typhi* cell division activator protein (StCAP) [34], as a novel inhibitor of TgSABP1. Molecular modeling suggests that eltrombopag competitively occupies the 6′SL-binding pocket and effectively blocks the interaction of TgSABP1 and 6′SL-PAA. This competitive binding results in significant suppression of *T. gondii* host cell invasion without affecting intracellular parasite replication at the concentration of 10 μM eltrombopag (Fig. 6D). These results confirm that eltrombopag’s anti-invasion mechanism derives from specific TgSABP1 targeting rather than general toxicity. However, eltrombopag did not show any anti-parasite activity in vivo, structural optimization may be needed for improvement of its affinity and accessibility to the parasite.

## Conclusions

This study characterizes a conserved globular domain presented across apicomplexan parasites as a novel SA-binding module, which mediates the SA recognition of TgSABP1 and its orthologs in related pathogens. Through integrated structural and functional analyses, we determined the SA-binding pocket of TgSABP1 at the molecular level and validated that the compounds targeting this pocket effectively inhibit *T. gondii* host cell invasion. These findings provide a structural framework for anti-parasitic drug discovery through screening invasion-associated proteins, and offer molecular templates for designing pathogen-specific inhibitors targeting the conserved SA-binding domain.

## Supplementary Information

Additional file 1: Table S1. The primers used in this study. Figure S1. The predicted structures of TgSABP1 and homologous proteins by AlphaFold2. Figure S2. The purified recombinant proteins were analyzed by SDS-PAGE. Figure S3. The binding curves of recombinant proteins with 3′SL-PAA and 6′SL-PAA analyzed by BLI assay. Figure S4. Amino acid residues of TgSABP1 within a 3 Å range of the SA molecule were visualized using PyMOL software.

## Data Availability

The data supporting the conclusions of this article are included within the article

## Abbreviations

BLI: Bio-layer interferometry
DMSO: Dimethyl sulfoxide
DBL: Duffy binding-like
EBL: Erythrocyte binding-like
FBS: Fetal bovine serum
IPTG: Isopropyl β-D-1-thiogalactopyranoside
IMP1: Immune mapped protein 1
MAR: Micronemal adhesive repeat
MOI: Multiplicity of infection
PBST-BSA: PBS containing 0.5% Tween 20 and 0.1% BSA
PBS: Phosphate-buffered saline
SA: Sialic acid
SDS-PAGE: Sodium dodecyl sulphate–polyacrylamide gel electrophoresis
3′SL-PAA: Neu5Acα3′-Lac-Gly-PAA-biotin
6′SL-PAA: Neu5Acα6′-Lac-C2-PAA-biotin
