# Supplementary material for: A sialic acid-binding protein in Toxoplasma gondii contains a conserved globular domain in apicomplexan parasites
Source: Parasit Vectors. 2025 Jul 1;18:239. doi: 10.1186/s13071-025-06845-5 (PMC12210739; doi:10.1186/s13071-025-06845-5)
Supplement: Supplementary file 1 — Additional file 1: Table S1. The primers used in this study. Figure S1. The predicted structures of TgSABP1 and homologous proteins by AlphaFold2. Figure S2. The purified recombinant proteins were analyzed by SDS-PAGE. Figure S3. The binding curves of recombinant proteins with 3′SL-PAA and 6′SL-PAA analyzed by BLI assay. Figure S4. Amino acid residues of TgSABP1 within a 3 Å range of the SA molecule were visualized using PyMOL software. [file 13071_2025_6845_MOESM1_ESM.docx]

**Additional file 1**

**Table S1.** The primers used in this study.

| **Primers** | **Sequence (5’-3’)** | Used for |
| --- | --- | --- |
| PfIMP2-F | ATGTCAGAAGAAAAGG | To amplify coding sequence of *PfIMP2* |
| PfIMP2-R | TTAAAAGATATACTAGCTCCT |  |
| PbIMP2-F | ATGTCAGATGATAAAGGAGCTT | To amplify coding sequence of *PbIMP2* |
| PbIMP2-R | CTAGAAAGAAATACTAGCCCC |  |
| PfIMP1-F | ATGAAGAAAAAACCTCCAAAGATG | To amplify coding sequence of *PfIMP1* |
| PfIMP1-R | TCAGGACAAACTTAATAATGATCCA |  |
| TgIMP2.1-F | ATGGGACTACCTGATTGTTTGAG | To amplify coding sequence of *TgIMP2.1* |
| TgIMP2.1-R | CTACAGGGCCAGAGCAGCTCCG |  |
| TgSABP1-F | ATGTCTGGCAACAACCCGT | To amplify coding sequence o *TgSABP1* |
| TgSABP1-R | TCACTTCTTAGCTGGGACAAAG |  |
| TgIMP1-F | ATGGGAACCGTTTGCACGAAG | To amplify coding sequence of *TgIMP1* |
| TgIMP1-R | CTACGCATTGCTCTGTCCA |  |


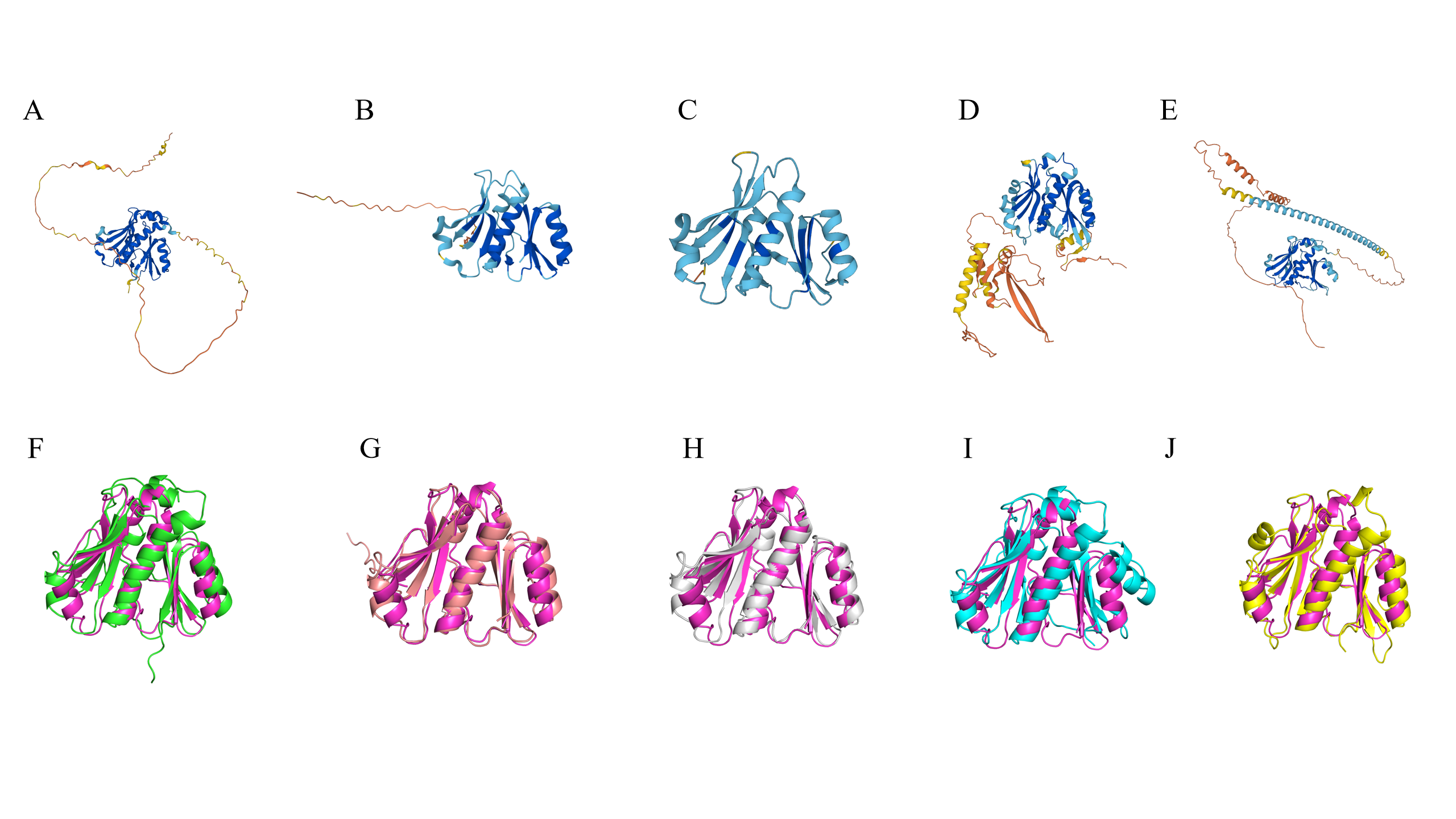


**Figure S1.** The 3D structures of TgSABP1 and homologous proteins were predicated by AlphaFold2 and structural overlapping by PyMOL (v2.5.4) software. The 3D structures of TgSABP1 (A: AF-S7UWV2-F1-v4), TgIMP2.1 (B: AF-A0A125YPT5-F1-v4), PbIMP2 (C: AF-A0A509AFF8-F1-v4), TgIMP1 (D: AF-A0A125YZ06-F1-v4), and PfIMP1(E: AF-Q8IJY0-F1-v4). AlphaFold2 produces a per-residue model confidence score (pLDDT) between 0 and 100. Some regions below 50 pLDDT may be unstructured in isolation. Dark blue indicates very high (pLDDT > 90); light blue indicates high confidence (90 > pLDDT > 70); yellow indicates low confidence (70 > pLDDT > 50); Orange indicates very low confidence (pLDDT < 50). Panels F-J show the globular structures of TgSABP1, TgIMP2.1, PbIMP2, PfIMP1. TgIMP1 is aligned to the structure of PfIMP2 using PoMOL and their RMSD values are 2.329 Å, 1.547 Å, 2.011 Å, 3.459 Å and 2.823 Å, respectively.


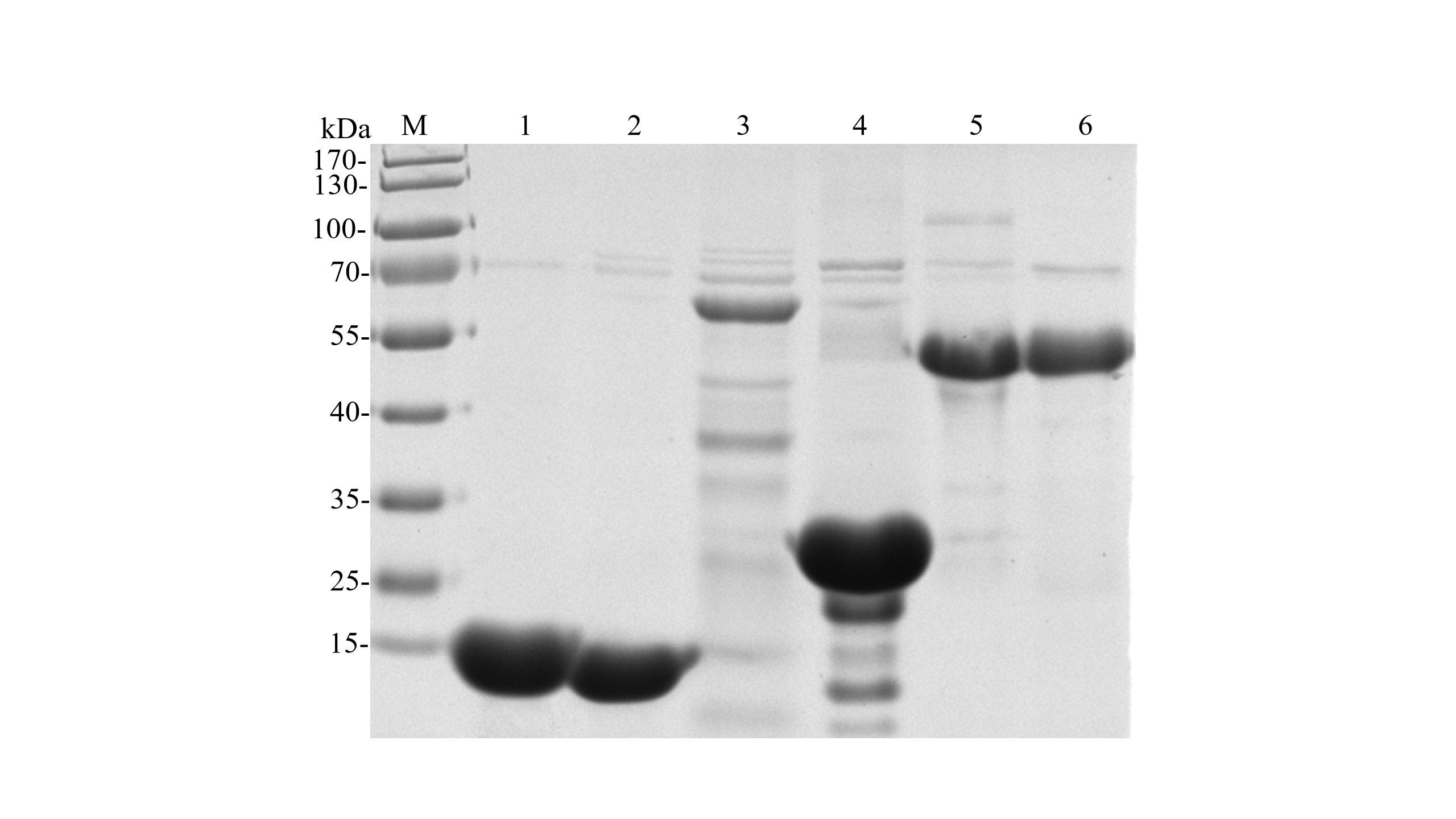


**Figure S2.** The purified recombinant proteins were analyzed using SDS-PAGE. Lane 1: His-tagged PfIMP2; Lane 2: His-tagged PbIMP2; Lane 3: His-tagged PfIMP1; Lane 4: His-tagged TgIMP2.1; Lane 5: His-tagged TgSABP1; Lane 6: His-tagged TgIMP1.

**
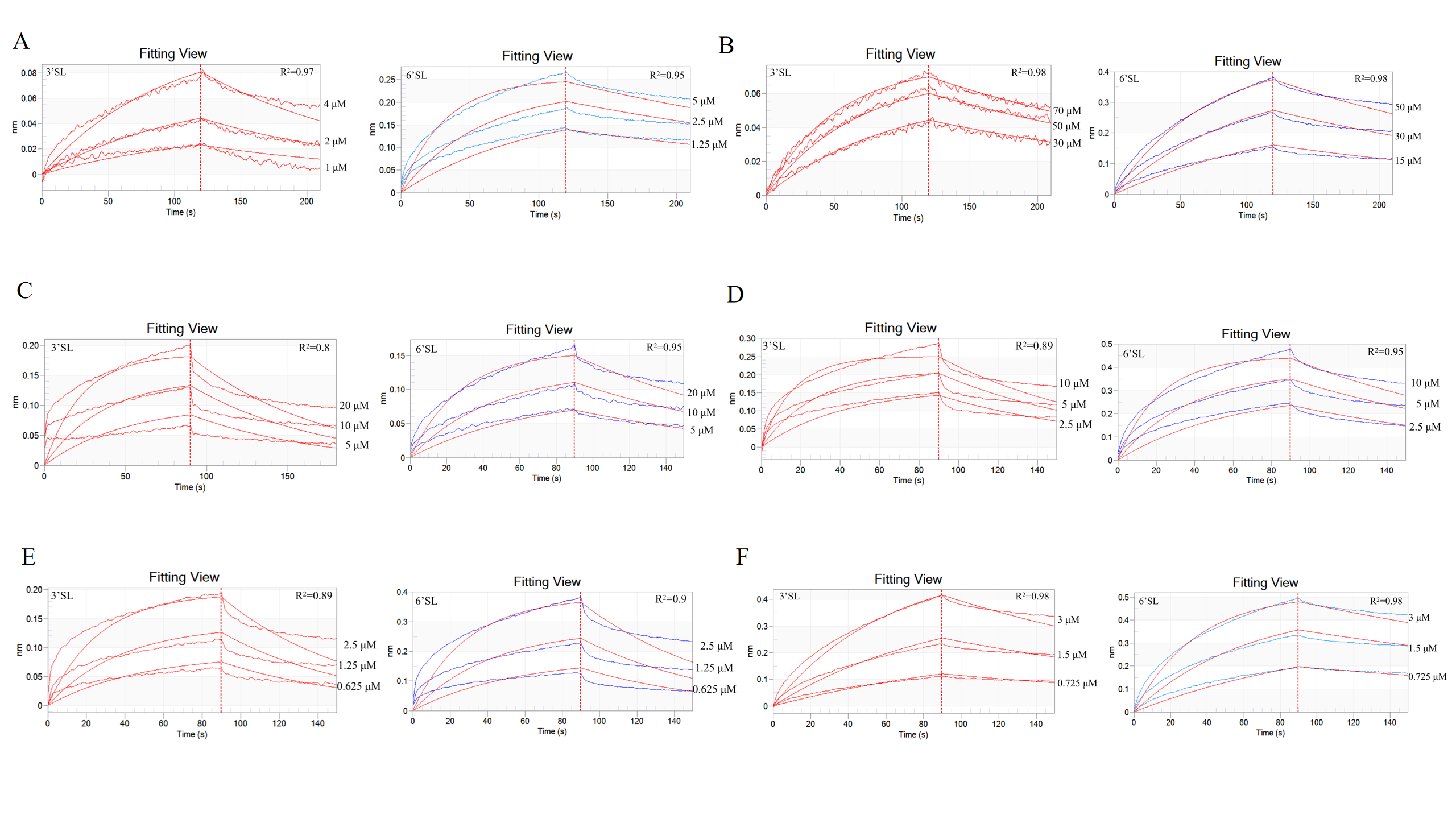
**

**Figure S3.** The binding curves of recombinant proteins with 3'SL-PAA (red line) and 6'SL-PAA (blue line) were analyzed by BLI. A: TgSABP1; B: PfIMP2; C: PbIMP2; D: PfIMP1; E: TgIMP2.1; F: TgIMP1.


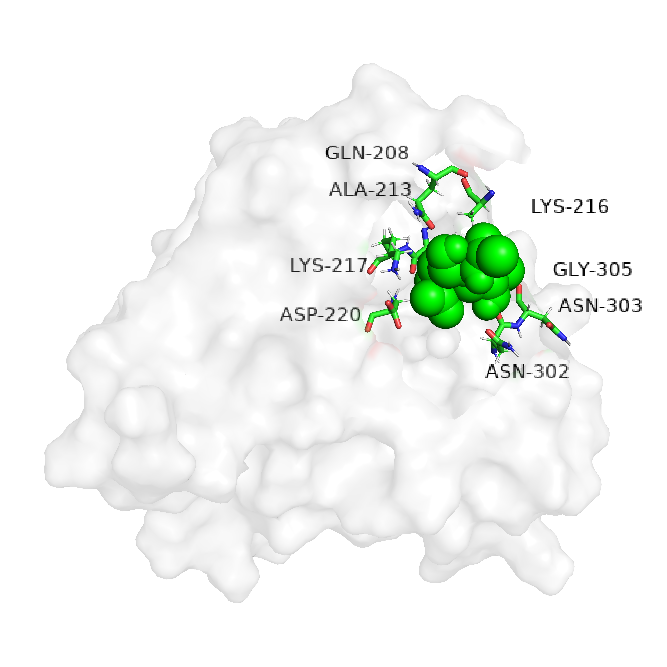


**Figure S4.** Amino acid residues within 3 Å of TgSABP1 potentially interact with SA were visualized using PyMOL software. SA is shown as green spheres, and the globular domain of TgSABP1 is shown as a light grey surface. Amino acid residues are shown as green sticks.
